# Canadian French translation and linguistic validation of a new patient-reported outcome measure for gender-affirming care: the GENDER-Q

**DOI:** 10.1186/s41687-026-01073-6

**Published:** 2026-08-05

**Authors:** Sylvie D. Cornacchi, Ines Ndzana Siani, Julia Sapin-Leduc, Flavie Guibert-Piché, Trisia Breitkopf, Charlene Rae, Hanya Obsieh, Manraj N. Kaur, Yi Wang, Alexis Laungani, Andrea L. Pusic, Anne F. Klassen

**Affiliations:** 1 Department of Pediatrics, McMaster University, Hamilton, Canada; 2 University of Quebec in Montreal, Montreal, Canada; 3 GrS Montréal, 999, rue De Salaberry, Montréal, Québec, H3L 1L2 Canada; 4 Patient-Reported Outcomes and Values, & Experience Center (PROVE), Mass General Brigham, Harvard Medical School, Boston, USA

## Abstract

**Background:**

The GENDER-Q is a patient-reported outcome measure (PROM) designed to assess outcomes and experiences of gender-affirming care from the patient’s perspective. The aim of this study was to translate the GENDER-Q into a linguistically validated Canadian French version.

**Methodology:**

We followed methods according to the Professional Society for Health Economics and Outcomes Research guidelines (formerly ISPOR) for PROM translation and cultural adaptation. This involved two forward translations, reconciliation, one back translation, review of the back translation, and cognitive debriefing interviews with individuals who identify as transgender and gender diverse (TGD).

**Results:**

The field test version of the GENDER-Q consisted of 55 scales with 959 items. This version was translated into Canadian French by two independent translators. The scales were back translated from Canadian French to English, then compared to the original English version to identify conceptual discrepancies. Based on the back translation, instructions for 13 scales, response options for 10 scales and 96 items required discussion and re-translation. Ten individuals who identified as TGD and were fluent in Canadian French reviewed the translated GENDER-Q via an online survey and subsequently participated in a cognitive debriefing interview to discuss scale components that, based on survey responses, were identified as difficult to understand. Participant feedback led to further re-wording of instructions, response options and items. The translation was reviewed to ensure consistency in the wording of recurring terms across the different scales.

**Conclusions:**

The GENDER-Q was translated and linguistically validated in Canadian French and can be used to inform gender-affirming care, clinical research, and quality improvement efforts.

**Supplementary Information:**

The online version contains supplementary material available at https://doi.org/10.1186/s41687-026-01073-6.

## Background

Patient-reported outcomes measures (PROMs) are standardized questionnaires designed to incorporate the patient’s voice into clinical care and research [1–4]. PROMs measure aspects of an individual’s health status, including symptoms, function, health-related quality of life (HRQL), and experiences of care [1–3]. PROMs can be used in research, value-based healthcare and quality improvement efforts to evaluate outcomes at health system, jurisdiction or population levels [5–7]. Within clinical practice, PROMs support individual patient care by evaluating treatment effectiveness, guiding shared treatment decision-making, facilitating patient-clinician communication, and identifying unmet needs or adverse outcomes [1, 5–7].

PROMs are being increasingly used to measure outcomes of different forms of gender-affirming care (GAC) in individuals who identify as transgender and gender diverse (TGD), including medical (e.g., gender-affirming hormones), surgical (e.g., face, voice, chest/breast, genitals), and mental health interventions [8–12]. Although some PROMs have been developed to measure specific domains of TGD people’s experiences (e.g., HRQL, urological or sexual function, gender incongruence, voice), most GAC research continues to use generic PROMs, instruments intended for use with cisgender populations and/or ad hoc questionnaires that lack validation or were not developed in accordance with international guidelines for PROM development [11–23]. Recent systematic reviews of PROMs used in GAC have underscored these limitations and the need for a rigorously developed, validated PROM that captures multi-dimensional aspects of individual’s experiences with GAC [15, 24–29].

To address this need, the GENDER-Q, a TGD-specific PROM was developed using established guidelines and best practices for PROM development and validation to assess outcomes and experiences of GAC [30–36]. Briefly, the GENDER-Q was developed from 84 concept elicitation interviews with TGD individuals from 4 countries (Canada, Denmark, the United States, and the Netherlands). Content validity was demonstrated through cognitive debriefing interviews with 14 TGD participants and feedback from 50 clinical experts [14]. A cross-sectional, international field-test study involving 5,497 TGD adults was conducted to perform item reduction, finalize the GENDER-Q scales and scoring algorithms and confirm reliability and validity [32]. The final version of the GENDER-Q includes 54 independently functioning scales and 2 symptom checklists covering 13 domains: health-related quality of life, sexual, urination, gender practices, voice, hair, face & neck, body, breasts, genital feminization, chest, genital masculinization, and experience of care [32].

The global uptake of PROMs in research and clinical care depends on their translation and cultural adaptation into multiple languages [36–41]. The accuracy of translated PROMs is critical to ensure they are inclusive, accessible, and use language that is sensitive and culturally appropriate [38, 40]. Poorly translated instructions and items may be misinterpreted by patients, thereby compromising a PROM’s reliability, validity, and the integrity of the data collected [39, 40]. Consequently, it is imperative that a rigorous, systematic approach be employed to develop high-quality translations. International guidelines recommend a multi-step process for PROM translation that includes forward and back translations, as well as feedback from the target patient population [40, 41].

The GENDER-Q has been translated into several languages (e.g., Danish, Dutch, German) [42, 43]. Canada has a large francophone population and is building capacity in GAC (e.g., 16,225 TGD individuals reside in Québec – 2021 population census) [44]. To enable the inclusion of Canadian French-speaking participants in the GENDER-Q field-test, the objective of this study was to translate the field test version of the GENDER-Q into a linguistically validated Canadian French version.

## Methods

We followed a multi-step process as described by the Professional Society for Health Economics and Outcomes Research (formerly ISPOR) and best practices for translation and cross-cultural adaptation of PROMs to translate the GENDER-Q into Canadian French [40, 41].

### Translation and cultural adaptation process

**Fig. 1:**
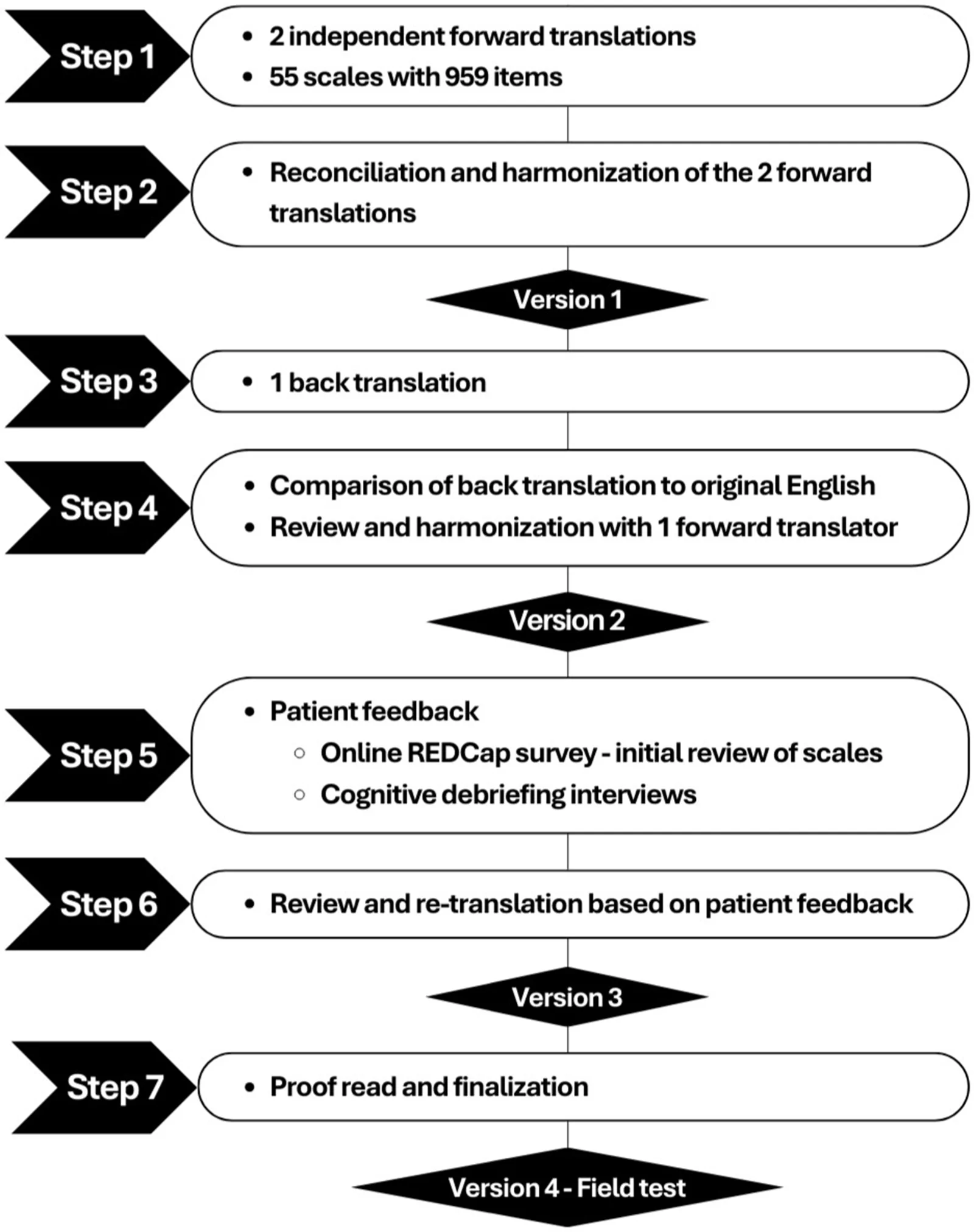
Steps in translation process

Our translation process is outlined in the 7 steps (Fig. 1) described below. Supplementary File – Appendix 1 includes a blank template of the Excel worksheet used to collect and analyze the data for all stages of the translation process.

#### Step 1 - Forward translation (English to French)

The field-test version of the GENDER-Q translated into Canadian French consisted of 55 scales with 959 items [31, 32]. Two native Canadian French speakers who are fluent in English (J.S.L., F.G.P.), independently translated the GENDER-Q scales.

#### Step 2 – Reconciliation and harmonization of forward translation

The translators met to review their translations, establish consensus, and create a single document (French Version 1).

#### Step 3 - Back translation (French to English)

Two native English speakers who are fluent in Canadian French (H.O.) and European French (I.N.S) produced a back translation. Each translator independently reviewed the French translation for half of the scales. Neither translator had access to the original English version of the GENDER-Q.

#### Step 4 - Back translation review

The goal of this step was to identify mistranslations or inaccuracies in French Version 1. Two members of the GENDER-Q research team who are native English speakers (Y.W.) with one also fluent in Canadian French (S.D.C.) independently reviewed the full back translation to identify discrepancies between the back translation and the English version. Discrepancies were discussed in a meeting with one of the original forward translators [author initials de-identified], who re-translated any parts where necessary (French Version 2).

#### Step 5 – Patient feedback

The goal of this step was to obtain feedback from patients on the translation (French Version 2). An online survey and follow-up cognitive debriefing (CD) interviews were conducted to determine if the translation was easy to understand and used accurate and appropriate wording.

#### Patient recruitment

Ethics approval was obtained from the Hamilton Integrated Research Ethics Board (#4718). Patients were eligible to participate if they were 18 years or older, were native Canadian French speakers, identified as TGD and had undergone GA surgery within the past five years. Patients were recruited through a private, high volume surgical clinic in Québec. An email invitation was sent to eligible patients by a member of their care team. Individuals who expressed a willingness to participate by return email were contacted by the interviewer (I.N.S.) to discuss the study and schedule an interview. Participants provided informed consent prior to their participation.

#### GENDER-Q scale review

**Fig. 2:**
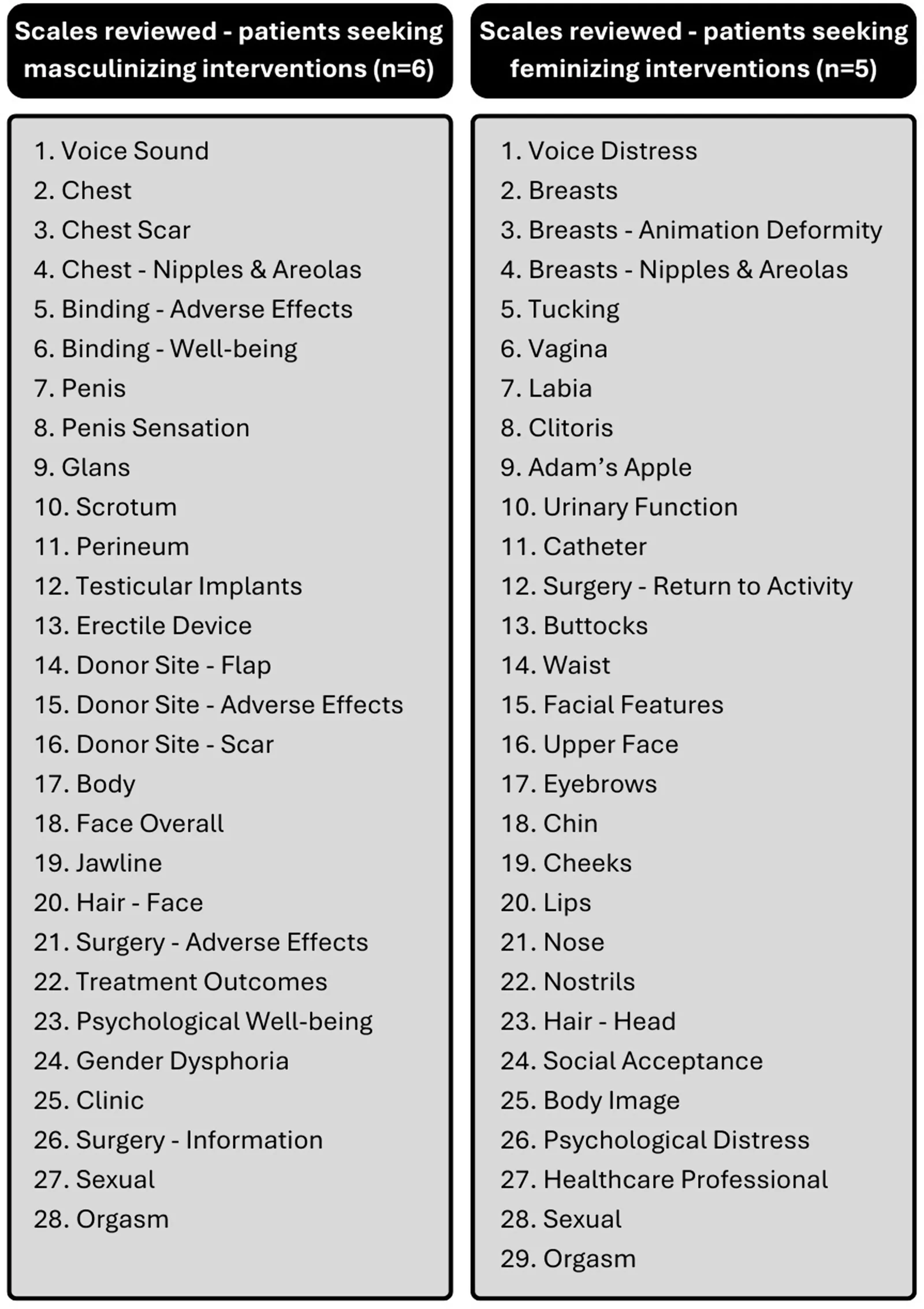
List of scales reviewed during online survey and cognitive debriefing interviews (*N* = 10). One participant who identified as gender fluid reviewed all the scales

Due to the large number of scales and items (55 scales, 959 items), a 2-step review process was used to reduce the burden on participants in advance of the CD interview:

1. **Online survey.** All participants were invited to complete a REDCap survey [45]. Participants were asked to read through scales related to their treatment goal. Specifically, participants who had feminizing treatments completed feminizing treatment scales (e.g., Breast, Vagina), and participants who had masculinizing treatments completed masculinizing treatment scales (e.g., Chest, Penis). In addition, all participant read through the Sexual and Orgasm scales. The remaining scales were randomly divided evenly between the two groups (see Fig. 2). Participants were asked to read through each scale’s instructions, response options, and items and to indicate whether the translation was easy to understand and made sense in French (Yes/No). Survey data were downloaded into IBM SPSS Statistics for Windows, v.30 (IBM Corp., Armonk, N.Y., USA) and analysed descriptively to identify content deemed difficult to understand for further review in the CD interviews.

2) **Cognitive debriefing (CD) interviews**. After completing the online survey, participants were invited to take part in a CD interview conducted via Zoom [Version 5.8.4]. These interviews followed a “think aloud” approach to explore how participants interpreted and understood the scale components, offering insight into their thought processes [46, 47]. During the interview, participants were asked to verbalize their thoughts as they read through the scales and to comment on words or phrases they found awkward or unclear. Items, instructions and response options marked as difficult to understand in the online survey were revisited (Supplementary File – Appendix 2 Cognitive Debriefing Interview Guide). Scales with missing survey responses were also reviewed to determine whether comprehension challenges contributed to nonresponse. Additionally, the interviewer probed for feedback on items that the translation team had identified as difficult to translate in the forward and back translation stages. Participants were encouraged to suggest alternative wording or phrasing that more accurately reflected their understanding and everyday use of Canadian French. Any proposed revisions were verified in later CD interviews to ensure consistency and acceptability across participants. Special attention was given to the translation of TGD-specific terminology, such as “binding” and “tucking”, to confirm conceptual equivalence and cultural appropriateness. Each GENDER-Q scale was reviewed by at least five participants, in accordance with translation guidelines [40]. During the interviews, the interviewer took detailed notes on participant feedback and suggested revisions. All interviews were audio-recorded and reviewed by a second bilingual team member [author initials de-identified], fluent in English and Canadian French, who documented additional observations. A $100 CAD gift card was provided to each participant as a thank-you upon interview completion.

#### Step 6 – Post-cognitive debriefing interview revisions

Both sets of notes from the CD interviews were reviewed independently by two team members (I.N.S, S.D.C) . Consensus was reached through discussion on all translation modifications resulting in French Version 3 of the GENDER-Q.

#### Step 7 – Proofreading and finalization

Version 3 was proofread for spelling and grammatical accuracy, and to ensure consistency in the wording of similar instructions, response options and, items across scales. This step resulted in the Canadian French Version 4 of the GENDER-Q (field test version).

## Results

### Translation and cultural adaptation process

The steps in the translation process and examples of changes made during each step are outlined below.

### Steps 1 – Forward translation

Between October and December 2022, the 55 GENDER-Q scales were independently forward translated from English to Canadian French by two translators who were native French-speakers and fluent in English.

### Step 2 – Forward translation reconciliation

The translators met to review the two forward translations and identified inconsistencies. The forward translators agreed on the translation of most items, with only minor differences in wording and phrasing identified between the two versions. These discrepancies were discussed by the translators and resolved through reconciliation. For example, for the English item “Explained treatment options clearly?”, one translator used the French phrase “*Vous a clairement expliqué les options de traitement?* (English: Clearly explained treatment options to you?) while the second translator chose the French phrase “*Vous a expliqué correctement les options de traitement?” (*English: Explained treatment options to you correctly?). Since “clearly” and “correctly” are conceptually different, the first option was chosen as it was closer in meaning to the English item.

Challenges were encountered in translating TGD-specific terminology into French, particularly for expressions such as the practice of “binding” and references to the “chest” in the scales assessing outcomes related to masculinizing chest surgery. For example, the French word *“torse”* (English: torso) was selected instead of “*poitrine*” (English: chest) as the word “*poitrine”* in French is commonly associated with breasts and therefore considered less appropriate for trans men and nonbinary individuals. The use of *“torse”* was confirmed to be more suitable during CD interviews with TGD participants. Table 1 provides additional examples of translation discrepancies and strategies used by the forward translators to achieve consensus.

**Table 1:** Examples of forward translation development and consolidation

GENDER-Q Scale	Item no.	Item (English)	Forward Translation 1*	Forward Translation 2*	Commentary and Consolidation*
Psychological Distress	9	Like giving up	French: ‘*Sur le point d’abandonner’* English: ‘About to give up’	French: *‘Découragé’* English: ‘Discouraged’	‘about to give up’ and ‘discouraged’ are conceptually different Selected Forward Translation 1 version as it was closer in meaning to the original item
Binding - Wellbeing	9	I feel relaxed around other people.	French: ‘*Je me sens détendue en présence d’autres personnes.’* English: ‘I feel relaxed in the presence of other people.’	French: *‘Je suis à l’aise avec les gens.’* English: ‘I’m at ease/comfortable with people.’	‘I feel relaxed’ and “I’m at ease/comfortable” are conceptually different Selected Forward Translation 1 version as the meaning was conceptually closer to the original item
Labia	12	How distinct your inner and outer labia are from each other?	French: ‘*La distance retrouvé entre vos grandes lèvres et vos petites lèvres?’* English: ‘The distance between your inner and outer labia?’	French: *‘À quel point vos grandes lèvres et vos petites lèvres sont distinctes?’* English: ‘How distinct are your inner and outer labia?’	‘the distance between’ and ‘how distinct’ are conceptually different Selected Forward Translation 2 version as it was closer in meaning to the original item
Breasts	19	How much cleavage you have when you wear a bra?	French: ‘*Votre décolté lorsque vous portez une brassière?’* English: ‘Your cleavage when you wear a bar?’	French: ‘*L’importance de votre décolleté lorsque vous portez un soutien-gorge?’* English: ‘The importance of cleavage when wearing a bra?’	*Revision*: ‘*La profondeur de votre décolleté lorsque vous portez un soutien-gorge?*’ English: ‘The depth of your cleavage when wearing a bra?’

*French phrases and items are *italicized*

### Steps 3 – Back translation

Two team members back translated the Canadian French Version 1 back into English, with each reviewing half the scales.

### Step 4 – Back translation review

The back translation review prompted revisions to the instructions for 13 scales, the response options for 10 scales and the wording of 96 items that were found to differ in meaning or to omit elements present in the original English version. These components were re-worded to improve conceptual equivalence, cultural appropriateness, completeness of meaning, and to ensure consistent definitions across scales. For example, the term “donor area” used in the Penis and Donor Site scales in reference to phalloplasty was re-translated from “*la zone de votre corps*” (English: area of your body) to “*la zone donneuse*” (English: donor area). Similarly, a response option in the Surgery – Return to Activity scale was modified from “*assez difficile*” (English: quite/rather difficult) to “*quelque peu difficile*”, which was closer in meaning to the English response option (English: somewhat difficult). Wording for similar items and instructions across scales was standardized for consistency. Some examples of items that were re-translated during this Step are shown in Table 2.

**Table 2:** Examples of challenges and revisions throughout the translation process (back translation review and cognitive debriefing interviews)

GENDER-Q Scale	Item no.	Item (English)	Forward Translation (French**)	Back Translation Review**	Cognitive Debriefing** Interviews
Scrotum	3	The location of your scrotum on your body **(not too far forward or back)?**	Translation: ‘… **(*pas trop haut ou trop bas)****’*	Back translation: ‘… (not too high or low)’ *Revision*: ‘***pas trop en avant ou en arrière***’ [English: ‘… (not too forward or back)’]	No change
Surgery - Adverse Effects	22	**Pulling sensation**	Translation: ‘*Sensation de tension’*	Back translation: ‘Feeling of tension’ *Revision*: ‘***Sensation de tiraillement’*** [English: ‘tugging/pulling sensation’]	No change
Facial Hair	1	How easy is it to **remove** facial hair (e.g., shave, pluck)?	Translation: ‘*Votre facilité à* ***entretenir*** *vos poils (p.ex.*,* raser*,* épiler)?’*	Back translation: ‘How easy it is to **groom/maintain** your facial hair (example shaving, plucking)?’ *Revision*: *‘…****enlever*** *les poils sur votre visage’* [English: ‘remove’]	No change
Chest	4	How well both sides of your chest match (look the same)? *	Translation: ‘*L’apparence* ***identique*** *des deux côtés de votre torse?’*	Back translation: ‘How similar both sides of your chest look?’ No change	*Revision*: *‘À quel point les deux côtés de votre torse sont* ***symétriques (ont la même apparence)?’*** [English: ‘How symmetrical are the two sides of your chest/torso (look the same)?’] was preferred by participants
Nose	10	The size of your nose **in relation to** your face?	Translation: *‘…****en relation avec’***	Back translation: **‘…in relation to’** No change	*Revision*: ***‘par rapport à’*** [English: the phrase ‘in relation to’ or ‘with respect to’] was preferred by participants
Urinary Function	16	My pee comes out like a **spray.**	Translation: ‘*Mon urine sort en* ***“spray.“****’* Used English term “spray” in quotes	Back translation: ‘My urine comes out as a spray.’ No change	*Revision*: *‘…sort en* ***éventaille (“spray”)***’ [English: ‘…comes out in a fan (“*spray*”).’] was preferred by participants
Breasts	22	How the scars from your **breast surgery** look?	Translation: ‘*L’apparence de vos cicatrices de chirurgie?’*	Back translation: ‘The appearance of your surgical scars?’ The ‘*breast*’ element of item was missing *Revision*: ‘…***sur vos seins****?’* was added [English: ‘…on your breasts?’]	No change
Return to Activity	14	Do **moderate** exercise (e.g., go for a **brisk walk**)?	Translation: *‘Faire des exercices* ***légers (marche****)?’*	Back translation: ‘Perform light exercise (walking)?’ *Revision*: *‘Faire des exercices* ***modérés*** *(p. ex.*, ***une marche rapide****)?’* [English: ‘Do moderate exercise (eg, a brisk walk)?’]	No change
Chest	2	How your chest looks in a loose **T-shirt**?	Translation: *‘L’apparence de votre torse dans un* ***T-shirt*** *ample?’* Used the English term “T-shirt”	Back translation: ‘The look of your chest in a loose-fitting T-shirt?’ No change	*Revision*: *‘L’apparence de votre torse dans un* ***chandail à manche courte (“t-shirt”)*** …*?’* [English: short-sleeved shirt] was preferred by participants

**Bold** words indicate the words or phrases that were challenging to translate

*Multiple scales included an item with this phrase

**French phrases and items are *italicized*

### Step 5 – Patient feedback and step 6 – post-cognitive interview revisions

Invitations were sent to 112 eligible TGD patients from the surgical clinic, of whom 10 consented and participated in both the online survey and CD interviews conducted between July and October 2023. Table 3 outlines participant characteristics. Five participants identified as a transman/man, four identified as a transwoman/woman and one participant identified as gender fluid. Participants ranged in age from 20 to 62 years. Nine participants were receiving hormone therapy, and all had undergone at least one form of GA surgery. The participant who identified as gender fluid reviewed all the scales. CD interviews lasted an average of 40 min (range 12–76 min). Summary tables of the REDCap survey results are provided in Table 4 (scale items) and Supplementary File – Appendix 3 (instructions and response options).

**Table 3:** Online survey and cognitive debriefing interview participant characteristics

Participant characteristics	*N* = 10
**Gender Identity**	
Transman/man Transwoman/woman Gender fluid	5 4 1
**Age (Years)**	
20–29 30–49 50–69	6 1 3
**Type of Gender-affirming Treatment**	
Hormone therapy Voice therapy Hair removal (face, body) Mastectomy Breast augmentation Phalloplasty or metoidioplasty Vaginoplasty	9 2 3 6 1 2 4

**Table 4:** Online REDCap survey results – Participant review of scale items

Scales (field test version) (*N* = 55)		Items					
		# Items (field test version)	# Raters	Raters x Items	# Ratings UNDERSTAND – NO		# Items UNDERSTAND – NO*
					*N*	%	*N*
Reviewed by participants seeking masculinizing interventions	Voice Sound	19	6	114	18	15.8	14
	Chest	17	6	102	10	9.8	6
	Chest Scar	19	6	114	2	1.7	2
	Chest – Nipples & Areolas	15	6	90	6	6.7	4
	Binding – Adverse Effects	21	6	126	7	5.6	7
	Binding – Well-being	12	6	72	0	0.0	0
	Penis	33	6	198	8	4.0	8
	Penis Sensation	16	6	96	5	5.2	3
	Glans	15	6	88^+^	2	2.3	2
	Scrotum	25	6	145^+^	12	8.3	10
	Perineum	14	6	81^+^	6	7.4	6
	Testicular Implants	14	6	81^+^	8	9.9	7
	Erectile Device	19	6	112^+^	11	9.8	9
	Donor Site – Forearm or Thigh	14	6	82^+^	2	2.4	2
	Donor Site – Adverse effects	13	6	78	1	1.3	1
	Donor Scar	18	6	108	4	3.7	3
	Body	12	6	72	3	4.2	2
	Face Overall	18	6	108	3	2.8	3
	Jawline	12	6	72	1	1.4	1
	Hair – Face	21	6	126	6	4.8	6
	Surgery – Adverse Effects	30	6	180	3	1.7	3
	Treatment Outcome	16	6	96	0	0.0	0
	Psychological Well-being	21	6	126	0	0.0	0
	Gender Dysphoria	16	6	96	2	2.1	2
	Clinic	19	6	114	3	2.6	3
	Surgery – Information	26	6	156	10	6.4	9
Reviewed by participants seeking feminizing interventions	Voice Distress	13	5	65	7	10.8	6
	Breasts	23	5	114^+^	18	15.8	13
	Breasts – Animation Deformity	12	5	60	20	33.3	11
	Breasts – Nipples & Areolas	13	5	65	5	7.7	4
	Tucking	15	5	74^+^	9	12.1	8
	Vagina	21	5	103^+^	21	20.4	13
	Labia	28	5	140	8	5.7	8
	Clitoris	17	5	84^+^	11	13.1	8
	Adam’s Apple	16	5	80	5	6.3	5
	Urinary Function	25	5	125	11	8.8	6
	Catheter	15	5	74^+^	0	0.0	0
	Surgery – Return to Activity	20	5	100	1	1.0	1
	Buttocks	11	5	55	4	7.3	4
	Waist	8	5	39^+^	5	12.8	5
	Facial Features	12	5	60	0	0.0	0
	Upper Face	15	5	75	3	4.0	3
	Eyebrows	6	5	29^+^	2	6.9	2
	Chin	13	5	65	5	7.7	5
	Cheeks	15	5	74^+^	4	5.4	4
	Lips	24	5	120	7	5.8	7
	Nose	18	5	89^+^	7	7.9	5
	Nostrils	10	5	50	3	6.0	3
	Hair – Head	18	5	90	12	13.3	11
	Social Acceptance	20	5	100	3	3.0	3
	Body Image	14	5	70	1	1.4	1
	Psychological Distress	19	5	95	0	0.0	0
	Healthcare Professional	33	5	165	4	2.4	4
Reviewed by all participants	Sexual	16	10	160	4	2.5	4
	Orgasm	14	10	140	1	0.7	1
	Total	959	10^**^	5393	314	5.8	258

*Number of items in the scale that were marked as difficult to understand **by at least one participant**

** 1 participant who identified as gender fluid reviewed all scales

^+^ A small number of items had no rating (missing data)

#### Instructions

In the survey, the instructions for 47 scales (85%) were identified as difficult to understand, with most flagged by only one or two participants. During the CD interviews, it became evident that the phrase “*En gardant à l’esprit de votre [partie du corps] …”* (English: with your [body part] in mind…) was unclear and redundant with the opening sentence of the instructions. Consequently, this phrase was removed from the instructions of 34 scales. A minor wording change was also made to the time frame “past week” which was changed from “*dernière semaine*” to *“semaine dernière*” and applied to 17 scales to better reflect common French usage. Wording changes were also made to the instructions of eight scales to improve clarity or to update terminology. Terms such as “labia”, “hairline”, “binding”, and “erectile device” were re-translated. Corresponding updates were made to the relevant items within these scales (32 items revised).

#### Response options

In the survey, the response options for 16 scales (29%) were marked as difficult to understand (Frequency *n* = 1; Difficulty *n* = 1; Bothered *n* = 1; Agreement *n* = 2; Amount *n* = 1; and Satisfaction *n* = 10). The majority (88%) of these were flagged by one participant. For the scales using Satisfaction, one participant noted grammatical inconsistency in the use of both feminine and masculine verb forms, suggesting that only the feminine form be retained in scales measuring outcomes of feminizing interventions. Based on CD interviews, one or more levels of the “bothered”, “agreement”, and “difficulty” response options were modified, resulting in updates to 8 scales. For example, in three scales using “agreement” response option, “*surtout en désaccord/d’accord*” (English: especially disagree/agree) was changed to “*modérément en désaccord/d’accord*” (English: mostly disagree/agree) based on participant preference.

#### Items

Table 4 provides a summary of the REDCap survey results for scale items. Of the 5,393 ratings of scale items completed by participants during the online survey, only 314 (5.8%) were marked as difficult to understand. Participants marked 258 different items (27%) as difficult with most items flagged by one (*n* = 231; 82%) or two (*n* = 38; 15%) participants. Only 9 items (3%) were identified as difficult by 3 or more participants. We modified an additional 74 items based on participant suggestions and preferences discussed during CD interviews.

Examples of revisions to scale components are summarized below and in Table 2:

- Translation of “bothered”: For the English response options, e.g., “not at all bothered, a little bothered…”, several French words have a similar but not exact meaning to “bothered”, e.g., “annoyed”, “troubled” or “disturbed”. The forward translators initially selected *“embêté”* (English: annoyed). However, following back translation review, *“dérangé”* (English: disturbed or troubled) was chosen for its closer semantic alignment with the intended English meaning. During CD interview discussions, participants preferred the term *“dérangé”.*
- Translation of “aligns with your gender”: For the English item “How well the appearance of your [body part] *aligns* with your gender?”, the two forward translators had used different expressions across scales, i.e., “aligns”, “matches”, or “corresponds”. After discussion, the translation “*s’aligne avec votre genre”* was initially selected (English: aligns with your gender). During CD interviews, participants were then asked to compare the alternatives, and the preferred wording was *“correspond à votre genre”* (English: corresponds with your gender) which was subsequently adopted across all relevant scales (*n* = 16).

### Step 7 – Proofreading and finalization

All 55 scales were proofread in detail to correct grammatical and typographical errors and to ensure consistent formatting (e.g., proper use of abbreviations such as *“p. ex.”* for “for example”). Items were also reviewed to ensure that both masculine and feminine verb forms were used appropriately throughout the scales, maintaining gender inclusivity (e.g., *“****·e****”* was added to the verb to designate the feminine verb form in addition to the masculine, “*Le son de votre voix lorsque vous êtes excité****·e****”*). These final refinements resulted in a linguistically accurate and inclusive Canadian French field-test version of the GENDER-Q (Version 4).

## Discussion

Adapting PROMs for use in different languages and cultural contexts can be a time-consuming and complex process. However, it is an essential step to ensure that translated instruments are inclusive, culturally meaningful and comparable when used in international settings (e.g., when pooling data across countries). Such adaptation increases the validity and generalizability of study results while reducing selection biases such as the exclusion of non-English-speaking participants from clinical studies [36–41]. The GENDER-Q is a comprehensive PROM that was designed to measure outcomes such as HRQL before and after receiving GAC, satisfaction with appearance, and experience of care [31, 32]. The Canadian French version expands the accessibility of the GENDER-Q for international research and clinical evaluation of GAC outcomes and experiences of care and supports cross-linguistic comparability of PROM data.

In a recent review of the published/grey literature and outcome measure repositories, we identified four validated TGD-specific PROMs that have been translated into French: (1) two were translated into Canadian French – the Transgender Congruence Scale and a shorter, adapted version of the Gender Identity/Gender Dysphoria Questionnaire for Adolescents and Adults Scale available as a free online “quiz” for the general public, and (2) two were translated into European French for use in France and Belgium – the Transsexual Voice Questionnaire (Male to Female) and the Operated Male-to-Female Sexual Function Index [48–51]. Of these, only the two European French translations reported adherence to internationally recognized translation guidelines [50, 51].

Our team followed international PROM translation and cultural adaptation guidelines [40, 41] to develop a conceptually equivalent Canadian French translation of the GENDER-Q. Items, instructions, and response options underwent an iterative, multi-step review that allowed us to re-translate challenging items or phrases and clarify definitions and instructions. Overall, TGD participants found the translated GENDER-Q scales to be easy to understand. Only 5.8% of over 5000 item ratings were flagged as difficult to understand.

During the translation process, it is important to obtain feedback from a diverse sample of participants who differ by relevant demographic and clinical variables that reflect the target population the scale will be used with [35, 36]. A strength of this study is the inclusion of a heterogeneous sample in the CD interviews, encompassing a broad age range (20–62 years), diverse gender identities, and a wide spectrum of treatments relevant to the scales being translated, including hormones, speech therapy, hair removal and different types of GA surgery. In addition, special attention was paid to ensure that TGD-related and GA terminology were translated and defined appropriately. As terminology related to TGD populations continues to evolve, the TGD-related terminology used in our scales may require periodic review and updating in the future. Another strength is that wording changes introduced in earlier CD interviews were incorporated into subsequent interviews to obtain feedback and achieve consensus on translation refinements. Redundancy was reduced by removing unclear and repetitive instructions, with careful attention paid to natural language flow. Finally, the translated scales were thoroughly proofread to ensure that recurring components, such as instructions and response options, were translated consistently across all 55 scales.

There are some limitations to this study. The CD interviews were audio-recorded but not transcribed verbatim for pragmatic reasons. However, the interviewer and a second team member took detailed notes, which were then used to inform revisions to the scales. Although the sample size for the CD interviews was small, and participants were recruited from a single clinical site, at least 5 individuals reviewed each scale, consistent with the Professional Society for Health Economics and Outcomes Research translation guideline recommendations [40]. A challenge we faced in conducting this translation relates to the number of scales in the GENDER-Q. The GENDER-Q provides comprehensive coverage of important HRQL and experience of care domains in GAC and therefore has 54 independently functioning scales and 2 checklists (final version). While it may seem pragmatic to translate scales once they have been field tested and item reduced, our extensive experience points to important limitations of this approach [52–54]. For example, translators provide valuable information about the items and scales from their perspective that can be used in the final decisions made about which items to retain and drop. We found that the combination of an online survey in advance of the CD interviews was very helpful as it made it possible to reduce participant burden by focusing on problematic parts of each scale. However, this may have been less comprehensive than reviewing each item sequentially in a CD interview.

The current study is limited to the linguistic validation of the Canadian French translation of the GENDER-Q. Future research is needed to psychometrically validate this version in a French-Canadian population. In addition, this translation of the GENDER-Q is specific to francophone speakers from Canada. Cultural adaptation is needed for its use in other French-speaking populations (e.g., France, Belgium).

## Conclusions

The Canadian French translation of the GENDER-Q provides a validated instrument for evaluating and monitoring outcomes and experiences of GAC from the perspective of French-speaking patients. A comprehensive, multi-step translation process that incorporated feedback from TGD individuals was employed to produce a conceptually equivalent and culturally appropriate Canadian French version of the GENDER-Q. The final Canadian French version of the GENDER-Q is now available to license to use in research and clinical care (https://qportfolio.org/gender-q/) .

## Supplementary Information

Below is the link to the electronic supplementary material.

Supplementary Material 1

Supplementary Material 2

Supplementary Material 3

## Data Availability

Due to the sensitive nature of this work, no dataset will be made available to protect participant privacy.

## Abbreviations

CAD: Canadian dollars
CD: Cognitive debriefing
FT: Field–test
GAC: Gender–affirming care
GA: Gender–affirming
HRQL: Health–related quality of life
PROM: Patient–reported outcome measure
REDCap: Research Electronic Data Capture
TGD: Transgender and gender diverse
