# Supplementary material for: Canadian French translation and linguistic validation of a new patient-reported outcome measure for gender-affirming care: the GENDER-Q
Source: J Patient Rep Outcomes. 2026 Aug 5;10:200. doi: 10.1186/s41687-026-01073-6 (PMC13737304; doi:10.1186/s41687-026-01073-6)
Supplement: Supplementary file 1 [file 41687_2026_1073_MOESM2_ESM.docx]

**Supplementary File – Appendix 2**

**Box 1 - Cognitive Debriefing Interview Guide**

**Scale Review**

- **Instructions (repeat for each instruction that was reported to be difficult to understand from the online survey)**
  - What was difficult to understand about the instructions for this scale?
  - Is there a specific word or phrase that was difficult for you to understand? If yes, what is the word/phrase?
  - How can the translation of the instructions be improved so that you can understand them correctly?
    - e.g., do you have any suggestions for wording changes
- **Items (repeat for each item that was reported to be difficult to understand from online survey)**
  - What was difficult to understand about the item?
  - Is there a specific word or phrase that was difficult for you to understand? If yes, what is the word/phrase?
  - How can the translation of this item be improved so you can understand the item’s meaning correctly?
    - e.g., do you have any suggestions for wording changes
- **Response options (repeat for each response option that was reported to be difficult to understand from the online survey)**
  - What was difficult to understand about this response option?
  - Is there a specific word that was difficult for you to understand? If yes, what is the word?
  - How can the translation of the response option(s) be improved so that you can understand its meaning correctly?
    - e.g., do you have any suggestions for wording changes
