# Supplementary material for: Canadian French translation and linguistic validation of a new patient-reported outcome measure for gender-affirming care: the GENDER-Q
Source: J Patient Rep Outcomes. 2026 Aug 5;10:200. doi: 10.1186/s41687-026-01073-6 (PMC13737304; doi:10.1186/s41687-026-01073-6)
Supplement: Supplementary file 2 [file 41687_2026_1073_MOESM3_ESM.docx]

**Supplementary File – Appendix 3**

**Table 1. Online REDCap Survey Results – Participant review of scale instructions and response options**

| **Scales (Field test version)**  **(N=55)** | | **# Raters** | **Instructions** | **# Raters** | **Response options** |
| --- | --- | --- | --- | --- | --- |
|  |  |  | **UNDERSTAND – NO** |  | **UNDERSTAND – NO** |
|  |  |  | **N*** |  | **N*** |
| Reviewed by participants seeking masculinizing interventions | Voice Sound | 6 | 1 | 5 | 0 |
|  | Chest | 6 | 1 | 6 | 0 |
|  | Chest Scar | 6 | 1 | 6 | 0 |
|  | Chest – Nipples & Areolas | 6 | 1 | 6 | 0 |
|  | Binding – Adverse Effects | 6 | 2 | 6 | 0 |
|  | Binding – Well-being | 6 | 2 | 6 | 0 |
|  | Penis | 6 | 1 | 5 | 1 |
|  | Penis Sensation | 6 | 2 | 6 | 1 |
|  | Glans | 6 | 1 | 6 | 0 |
|  | Scrotum | 6 | 1 | 5 | 1 |
|  | Perineum | 6 | 1 | 5 | 1 |
|  | Testicular Implants | 6 | 1 | 6 | 0 |
|  | Erectile Device | 5 | 0 | 5 | 0 |
|  | Donor Site – Forearm or Thigh | 6 | 0 | 6 | 0 |
|  | Donor Site – Adverse effects | 6 | 3 | 5 | 0 |
|  | Donor Scar | 6 | 1 | 6 | 0 |
|  | Body | 6 | 0 | 6 | 0 |
|  | Face Overall | 6 | 0 | 6 | 0 |
|  | Jawline | 6 | 0 | 6 | 0 |
|  | Hair – Face | 5 | 1 | 6 | 1 |
|  | Surgery – Adverse Effects | 6 | 3 | 6 | 0 |
|  | Treatment Outcome | 6 | 0 | 6 | 1 |
|  | Psych Well-being | 6 | 2 | 6 | 0 |
|  | Gender Dysphoria | 6 | 1 | 6 | 0 |
|  | Clinic | 6 | 0 | 6 | 1 |
|  | Surgery – Information | 6 | 0 | 6 | 0 |
| Reviewed by participants seeking feminizing interventions | Voice Distress | 5 | 2 | 5 | 0 |
|  | Breasts | 4 | 2 | 4 | 1 |
|  | Breasts - Animation Deformity | 5 | 2 | 5 | 1 |
|  | Breasts – Nipples & Areolas | 5 | 2 | 5 | 1 |
|  | Tucking | 5 | 2 | 5 | 0 |
|  | Vagina | 5 | 2 | 5 | 1 |
|  | Labia | 5 | 2 | 4 | 1 |
|  | Clitoris | 4 | 2 | 4 | 1 |
|  | Adam’s Apple | 5 | 3 | 5 | 2 |
|  | Urinary Function | 5 | 1 | 5 | 0 |
|  | Catheter | 5 | 2 | 5 | 0 |
|  | Surgery – Return to Activity | 5 | 3 | 5 | 2 |
|  | Buttocks | 5 | 2 | 4 | 0 |
|  | Waist | 5 | 2 | 5 | 0 |
|  | Facial Features | 5 | 2 | 4 | 0 |
|  | Upper Face | 5 | 2 | 5 | 0 |
|  | Eyebrows | 5 | 2 | 5 | 0 |
|  | Chin | 5 | 2 | 5 | 0 |
|  | Cheeks | 5 | 2 | 5 | 0 |
|  | Lips | 5 | 2 | 5 | 0 |
|  | Nose | 5 | 2 | 5 | 0 |
|  | Nostrils | 5 | 2 | 5 | 0 |
|  | Hair – Head | 5 | 2 | 5 | 0 |
|  | Social Acceptance | 5 | 1 | 5 | 0 |
|  | Body Image | 5 | 1 | 5 | 0 |
|  | Psychological Distress | 5 | 1 | 5 | 1 |
|  | Healthcare Professional | 5 | 2 | 5 | 0 |
| Reviewed by all participants | Sexual | 10 | 2 | 10 | 0 |
|  | Orgasm | 10 | 4 | 10 | 0 |
|  | Total | 10** | 47 scales (85%) | 10** | 16 scales (29%) |

*Number of participants who reported the scale component was difficult to understand

**1 participant who identified as gender fluid reviewed all scales
